# Long Term Results of Modified Intersphincteric Resections for Low Rectal Cancer: A Single Center Experience

**DOI:** 10.3390/medicina55120764

**Published:** 2019-11-29

**Authors:** Vlad-Olimpiu Butiurca, Călin Molnar, Copotoiu Constantin, Marian Botoncea, Teodor Ioan Bud, Zsolt Kovacs, Cătălin Satala, Simona Gurzu

**Affiliations:** 1 First Department of Surgery, University of Medicine, Pharmacy, Science and Technology ‘George Emil Palade’, 540139 Târgu-Mureș, Romania; vladbutiurca@yahoo.com (V.-O.B.); chir1tgm@yahoo.com (C.C.); botonceam@gmail.com (M.B.); 2 Clinic of Vascular Surgery, Emergency County Hospital, 540139 Târgu-Mureș, Romania; theodor.bud@gmail.com; 3 Department of Pathology, Emergency County Hospital, 540136 Târgu-Mureș, Romania; kovacska_zsoltkovacs@yahoo.com (Z.K.); stlcatalin92@yahoo.com (C.S.); 4 Department of Pathology, University of Medicine, Pharmacy, Science and Technology, 540139 Târgu-Mureș, Romania; simonagurzu@yahoo.com

**Keywords:** intersphincteric resection, low rectal cancer, Wexner score, survival, functional outcome

## Abstract

Background and Objectives: The objective of this article is to evaluate the long-term oncological and functional outcomes following modified intersphincteric resections (ISR) for low rectal cancer. The modified technique consisted of the abandonment of colonic J-pouches, transverse coloplasty, or defunctioning temporary stoma in favor of a direct handsewn coloanal anastomosis (CAA). Material and Methods: Sixty consecutive patients with type II and III (juxta-anal or intra-anal) low rectal tumors underwent modified ISR by the same surgical team and were followed for a period of five years. Functional outcomes using the Wexner Score, postoperative complications, recurrence rates, morbidity, and mortality rates were assessed. Results: The five-year survival rate was 93.3% with a disease-free interval at three years of 98%. Morbidity was 15% (n = 9) consisting of intestinal wall necrosis (n = 6), stenosis (n = 2), and sacral metastasis (n = 1). The Wexner score values were, at 1 year, 8.5 (range, 4–13); at three years 7.2 (range, 2–11); and at 5 years 6.7 (range, 2–12). A second surgery was needed in only one case that showed postoperative transmural necrosis of the colonic wall. Conclusions: In highly selected patients with type II or III low rectal tumors and proper preoperative imaging staging, ISR might be a viable alternative to other techniques such as abdominoperineal resection and low anterior resection, both from a functional and an oncological perspective.

## 1. Introduction

Intersphincteric resection has long passed the status of a new procedure for low rectal cancer. Ever since Schiessel et al. [1] described the technique in 1994, surgeons have studied its benefits and pitfalls. With the introduction of sharp rectal dissection along the mesorectum (total mesorectal excision, TME) either from the abdomen or transanal TME (bottom-to-up approach), this procedural step in low rectal cancer surgery became the gold standard [2]. Radiotherapy and chemotherapy are in common use and can lead to downsizing and downstaging, making these adjuvant therapies suited to low rectal cancer [3,4]. Different guidelines, trials, and meta-analyses exploit the use of either preoperative chemotherapy and radiotherapy or radiotherapy alone [4,5], but a consensus on this matter has yet to be achieved. European Society for Medical Oncology (ESMO) guidelines recommend the use of risk-adapted treatment based on preoperative staging. This is done by employing either short course-preoperative radiotherapy (SCPRT) or long-course chemoradiotherapy (CRT) [6]. Imaging studies both prior to and after radiotherapy are of the utmost importance in determining the feasibility of ISR, identifying surgical planes, structure involvement, and discovering local recurrence [6,7,8].

Intersphincteric resections can be performed for patients with type II (juxta-anal) or type III (intra-anal) tumors for which partial intersphincteric resections (ISR) or total ISR is performed, respectively [9]. The technique incorporates a combined abdominal and perineal approach. After the primary vascular approach consisting of high ligation of the inferior mesenteric vessels is completed, TME down to the level of the pelvic floor follows [10]. The abdominal approach can be achieved using open surgery or laparoscopy. Subsequently, the perineal approach consists of internal anal sphincter (IAS) dissection. The distal resection line can be located at the level of the intersphincteric groove (for total ISR) or at the dentate line (for partial ISR). The specimen is usually delivered through the anus and is followed by a handsewn coloanal anastomosis (CAA). The original technique also featured the creation of either a colon J-pouch, transverse coloplasty, or defunctioning temporary stoma.

In this paper, we present a surgical team’s results using a modified version of the classic ISR, easily applied in daily practice. It consists of the abandonment of colonic J-pouches, transverse coloplasty, or defunctioning temporary stoma in favor of a CAA. The purpose of this study is to assess the long-term survival, and functional and oncological outcomes of intersphincteric resections.

## 2. Materials and Methods

### 2.1. Selection Criteria

The present study involved consecutive patients with low rectal cancer who underwent surgery in our department over a period of 5 years, between 2013–2018. This is a prospective study that was performed after obtaining the approval of the Ethics Committee of the University of Medicine, Pharmacy, Science and Technology ‘George Emil Palade’ of Târgu Mureș, and the Emergency County Hospital of Târgu-Mureș, Romania (nr. 330/17.11.2017, date of approval nr. 789/14.01.2016). All patients provided written informed consent prior to surgery.

We have included those patients with type II or III low rectal tumors who refused a colostomy or ileostomy [9]. All patients underwent long-term pelvic preoperative radiotherapy with a total dose of 50 Gy in 25 fractions [6]. Besides the patient’s decision, the following inclusion criteria were used: tumors located at 10–40 mm from the anal verge, 15 mm from the dentate line, or 10 mm from the anorectal ring. Another inclusion criterion was represented by adequate preoperative sphincter function and continence, objectified using the Wexner Score System and including only patients showing a score of ≤ 10 prior to surgery. A Wexner score evaluates the continence of the patient, giving insight on the status of the sphincteric apparatus (Table 1). On a scale of 0–20, 0 represents perfect continence, whilst 20 implies complete incontinence [11]. In the present study, only patients with preoperative Wexner score ≤ 10 were included.

### 2.2. Exclusion Criteria

**Figure 1:**
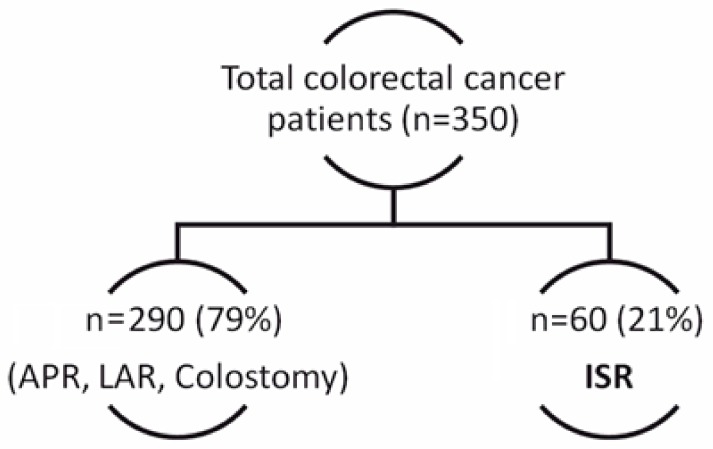
Selection of patients who underwent intersphincteric resection (ISR).

The following exclusion criteria were used: stage ≤ III B (American Joint Committee on Cancer (AJCC) Stage), post-radiotherapy Wexner Score ≤ 10, and MRI-proven fascia involvement. Based on the above-mentioned criteria, 60 patients with low rectal cancer have been selected for ISR (Figure 1) from 350 patients with colorectal cancer (left-sided colon cancer and rectal cancer) operated on in our institute between 2013–2018.

### 2.3. Diagnostic Management and Preoperative Staging

Based on current guidelines, the patients included (Table 2) in the study underwent preoperative colonoscopy with tumor biopsy and staging MRI in the majority of cases. Staging also involved the use of abdominal ultrasound, chest X-Ray, and computed tomography [6,12,13,14,15,16,17,18], and we depended on patient demographic data as our surgical center is a regional one and some patients were admitted with investigations ordered by other specialists. In some patients, emergency intervention was necessary, as severe rectorrhagia or incomplete bowel emptying was noted.

### 2.4. Surgical Technique

The majority of patients (n = 58) underwent open surgery, whilst two (n = 2) patients benefited from a combined laparoscopic/open approach. The technique consisted of first, an abdominal approach and, based on the concept of primary vascular approach and TME [19,20,21], the mobilization of the rectum down to the upper level of the levator ani muscle. The intersphincteric groove was assessed, when possible, to determine tumor invasion. The perineal assessment began with digital and instrumental dilation, followed by exposure of the anal canal, using four to six traction threads. After exposure, a circumferential incision was made on the anal mucosa distal to the dentate line (for total ISR) or at the level the dentate line (for partial ISR). A minimum distance of 1 cm distally was maintained in all cases. The perineal phase continued with intersphincteric circumferential cranial preparation to meet the dissection plane from the abdomen. Following completion of the dissection, the rectum was delivered through the anus, with transection of the sigmoid colon at the appropriate level. The final part of the surgery consisted of a hand-sewn coloanal anastomosis [22].

In all cases, colonic J-pouches, transverse coloplasty, or defunctioning temporary stoma were abandoned in favor of a direct handsewn coloanal anastomosis, which is the original element of this research.

### 2.5. Assessment and Follow-Up

**Figure 2:**
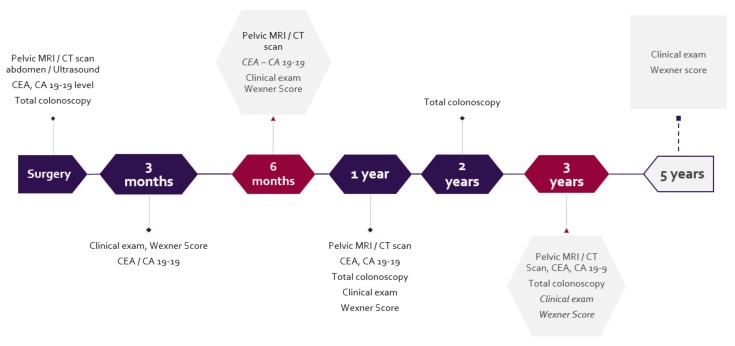
Follow-up protocol.

Follow-up was done in accordance with the timeline below (Figure 2). Patients with high-risk-stage tumors on the pathology report (angioinvasion, inadequate lymphadenectomy, tumor perforation) were submitted to the Oncologic Committee review for further management, consisting of additional chemotherapy in most cases.

Histopathology report quality parameters were reviewed to assess the completeness of mesorectal excision: completeness of mesorectal fascia, circumferential resection margin (CRM), and distal resection margin (DMR). DRM and CRM > 1 mm were considered to be negative [23]. Local recurrence was defined as any recurrence at the pelvic level. Distant metastasis was defined as any recurrence outside the pelvis. Postoperative complications, morbidity, and mortality rates were recorded. Wexner Score was determined for all patients on initial assessment, after preoperative radiotherapy, and at follow-ups (1, 3, and 5 years following surgery).

### 2.6. Statistical Assessment

Statistical analysis was performed using GraphPad, version 8.1.2 (San Diego, CA, USA) with a significance level of *p* = 0.05. using chi-squared test.

## 3. Results

### 3.1. Clinicopathological Characteristics and Operative Particularities

Patient background was in most cases urban (n = 43), with few patients from rural areas (n = 17). A personal history of cancer was found in seven (12%) patients but was not well documented, and a family history of cancer was noted in 12 (20%) patients.

Of the 60 patients, 47 benefited from partial ISR, and total ISR was performed in the other 13 cases (Table 2). Complete mesorectal excision, according to histopathology reports, was achieved in 54 cases (90%). CRM was negative in 57 (95%) of the cases.

### 3.2. Postoperative Complications

A total of nine (15%) patients developed postoperative complications. The commonest complication (n = 5) was the mucosa/submucosa necrosis of the pulled-through colon. This complication was noted at approximately 11 days following surgery. No second surgery was necessary for this complication. One patient developed postoperative pulled-through colon transmural necrosis, which required reintervention with reanastomosis. Stenosis of the coloanal anastomosis was found in two cases and local recurrence, represented by cutaneous sacral metastasis, occurred in another patient. Anastomotic leakage was not noted in any of the patients.

### 3.3. Overall Survival

Follow up was done every three months in the first year and at three and five years afterwards. One-year over-all and three years over-all survival was 100%. The disease-free interval, as objectified by MRI/CT scans, CEA/CA 19–19 serum levels, and the clinical exam, was 98% at three years following surgery. Median follow-up was 56.3 months with five-year overall survival of 93.3%. The mortality rate at five years was 7.3%.

### 3.4. Wexner Score

**Figure 3:**
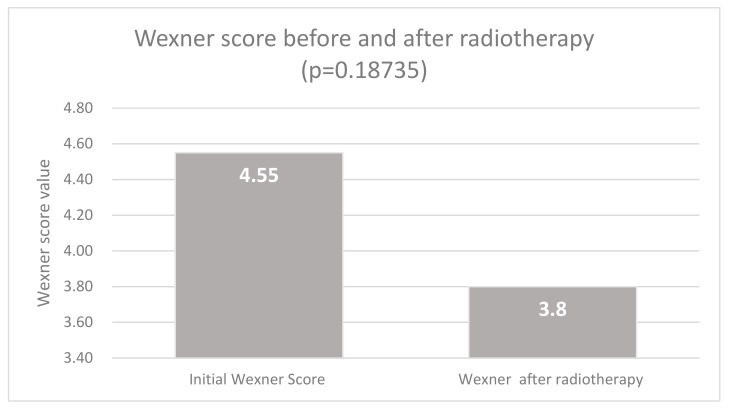
Values of Wexner score before and after radiotherapy.

The Wexner score showed a slight but not significant decrease after preoperative radiotherapy, compared with initial presentation (*p* = 0.18). The median value of the score, at initial presentation, was 4.55 ± 1.76 (range, 0–8). After radiotherapy, it decreased to a median value of 3.8 ± 1.85 (range, 0–7) (Figure 3).

**Figure 4:**
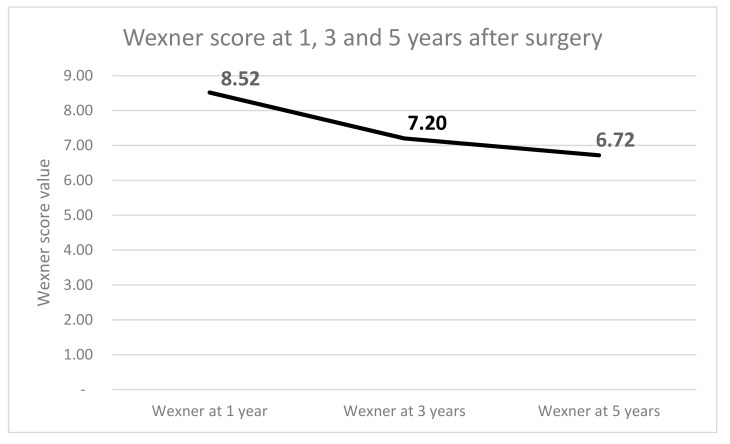
Values of Wexner score at 1, 3, and 5 years following surgery.

The postoperative Wexner score showed the following values: at 1 year, 8.55 ± 2.33 (range, 4–13), at three years, 7.25 ± 2.14 (range, 2–11) and at 5 years, 6.75 ± 2.12 (range, 2–12) (Figure 3). Significant decreasing of the Wexner score (Figure 4) was noted between one and five years postoperative follow-up (*p* = 0.000057; *t* = 4.17769).

## 4. Discussion

The study population included in the present paper comprised 60 patients followed over a period of five years. Considering that our service is not primarily a colorectal center, the size of the group is sufficient. Moreover, many studies have been reported based on sample sizes ranging from 19 to 80 patients [24,25,26,27].

When planning ISR for low rectal cancer, patient selection is paramount. There are numerous factors that affect both the feasibility of the procedure and the outcome. A clinical exam and a rectal touch performed by an experienced surgeon is the first step in both diagnosing and evaluating the feasibility of ISR. In these patients, a rigid proctosigmoidoscopy seems to localize the tumor better [28].

Another important step in the diagnostic and treatment selection algorithm is the preoperative MRI [12]. Specialized imaging is required to showcase the relationship between the tumor and the internal (IAS) and external anal sphincter (EAS) and allows the multidisciplinary team to establish the best management for each patient [12,13,14,15]. Imaging findings allow proper preoperative staging and can provide information regarding local recurrence risk [15]. In addition, MRI can be used to estimate the quality of CRM, with an overall accuracy of 88% [16,17]. Another important aspect in MRI-low-rectal-cancer staging is the relation of the mesorectal fascia to the tumor [18]. Imaging data, along with postradiotherapy sphincter function quality (Wexner score), guided us in choosing ISR as a treatment option. Due to our center being a regional one handling patients that have already been diagnosed in other medical services, our small study group is highly heterogenous regarding the best imaging diagnostic protocol for low rectal cancer. In our experience MRI gives the surgeon the best information regarding local anatomy, with tumor spread being the best tool to guide surgical decisions.

Another crucial factor in choosing ISR as a surgical option was patient refusal of a colostomy. Tumor type and location (at 1–4 cm from the anal verge) were also used for patient selection. All patients in our study showed confinement of the tumor to the rectal wall prior to surgical treatment, but ISR can also be performed for tumors staged as T_3_ and even T_4_ [9,24].

There is no consensus concerning the indication of this procedure in regard to tumor distance from the anal verge. Various authors reported different indications in both cohort studies and review studies ranging from > 4cm to > 6cm from the anal verge [23,29,30].

Our choice to perform a direct coloanal anastomosis (CAA) with the abandonment of coloplasty or rectal reservoir was based on personal experience. This aspect represents an ongoing debate; authors such as Spanos [31], Martin [32], Chen [27] and Shirouzu [29] reported that partial ISR (as opposed to total ISR) and colon J-pouches improve function in the first year after surgery, but the effect is not sustained after one year. Previous randomized studies advocate performing a pouch anastomosis to improve functional outcomes [33,34].

A 2018 review of the functional and oncological outcomes of ISR published by Park [24] and a meta-analysis by Fichera [35] show that there are minimal differences in functional outcomes between the pouch and straight CAA.

These results are probably due to ongoing technological, adjuvant therapy, and diagnostic improvements resulting in ISR being performed following targeted quality radiotherapy and in earlier stages of the disease yielding better functional results.

Our working hypothesis is based on the early use of the sphincterian apparatus and accessory muscles involved in continence by performing a direct CAA and abandonment of colonic J-pouches, transverse coloplasty, or defunctioning temporary stoma.

Morbidity and mortality rates found in our study are similar to those reported by other authors, as well as to overall survival [23,24,25,26,27,29,30,31,36].

In this paper Wexner score values, which are easy to quantify by both surgeon and patient, were used for choosing the best surgical option (ISR or APR) but also for follow-up. A significant decrease of this score was noted after long-term follow-up. Numerous studies have sought to discover the best score to use following rectal surgery, but a consensus has yet to be reached, particularly for evaluation of quality of life [37,38]. The Wexner score has established itself as a good instrument in diagnosing and grading fecal incontinence [39].

Various values of the Wexner score following ISR have been reported, showing a score of ≤ 12 at one and three years following surgery [23,29] or even lower in the Japanese experience [40]. In our study, the smallest median value of the Wexner score was 6.7 at five years. This is consistent with other reports; the difference in our study is probably due to our inclusion criteria. The difference between median pre-radiotherapy and preoperative Wexner scores (4.55 vs. 3.8) is due to downsizing, which is a well-documented aspect. On the other hand, radiotherapy doses negatively influence postoperative functional results [3,4,5,6], making the use of radiotherapy an ongoing debate from a functional point of view.

The negative aspect of the Wexner score, as previously stated, is its inability to assess quality of life directly. Quality of life can be assessed using various questionnaires, but all depend on numerous factors such as region, population background, etc. [39]. The Wexner score remains, in our opinion, the easiest to use for both surgeon and patient. A systemic review done by Ursi et al. [41] with data collected from 25 different studies reached similar conclusions to our own in regard to continence following ISR. Functional outcomes were influenced by neoadjuvant CRT but not necessarily by the type of surgery, as is the case in our study. Last, but not least, the authors of this review consider that patients choosing to avoid a permanent stoma are more inclined to accept the imperfect continence of ISR as opposed to the presence of a colostomy bag.

More than half of the patients in our study reported the acceptance of postoperative symptoms such as fecal incontinence, urgency, and fragmented bowel movements compared with having a colostomy bag.

## 5. Conclusions

Oncological surgical and functional outcomes following open ISR are acceptable. The technique is an already-established alternative to abdominoperineal resections in selected cases. Direct anastomosis following ISR with the abandonment of colonic reservoirs and defunctioning ostomies has good results. Evaluation of more long-term functional results and patient quality of life are needed.

## Figures and Tables

**Table 1:** Criteria of Wexner score assessment.

	Never	Rarely	Sometimes	Usually	Always
Solid	0	1	2	3	4
Liquid	0	1	2	3	4
Gas	0	1	2	3	4
Wears pad	0	1	2	3	4
Alters lifestyle	0	1	2	3	4

**Table 2:** Demographics, tumor characteristics, and type of intersphincteric resection (ISR).

Parameter	Value (n = 60)
Age (years)	67.32 ± 21.45 (range 57–81)
Male/female ratio	2.16:1
*Tumor characteristics*	
Distance from anal verge (cm)	3.15 ± 1.82 (range 1–4)
*Localization*	
Intra-anal (type III)	13 (22%)
Juxta-anal (type II)	47 (78%)
*Procedure*	
Partial ISR	47 (78%)
Total ISR	13 (22%)
*Serum markers*	
CEA > 12 ng/mL	63%
CA 19–9 > 900 U/mL	45%
